# Clean Your Cellars

**Published:** 1880-03

**Authors:** 


### CLEAN YOUR CELLARS.

By a beneficial arrangement of Providence, the gases and odors most prejudicial to human life, are lighter than the air which surrounds us, and, as soon as disengaged, rise immediately to the upper atmosphere, to be purified, and then returned to be used again.

The warmer the weather, the more rapidly are these gases generated, and the more rapidly do they rise, hence it is, that in the most miasmatic regions of the Tropics, the traveler can with safety pursue his journey at mid-day, but to do so in the cool of the evening, or morning, or midnight, would be certain death. Hence also the popular but too sweeping dread of "night air." To apply this scientific truth to practical life in reference to the cellars under our dwellings, is the object of this article.

The ceilings of cellars should be well plastered, in order most effectually to prevent the ascent of dampness and noisome odors through the joints of the flooring.

The bottom of the cellar should be well paved with stone, cobble stones are perhaps best; over this should be poured, to the extent of several inches in thickness, water lime cement, or such other material as is known to acquire in time almost the hardness of stone; this keeps the dampness of the earth, below.

If additional dryness is desired for special purposes, in parts of the cellar, let common scantling be laid down, at convenient distances, and loose boards be laid across them for convenience of removal and sweeping under, when cleaning time of the year comes.

The walls should be plastered, in order to prevent the dust from settling on the innumerable projections of a common stone wall.

Shelves should be arranged in the centre of the cellar, not in the corners, or against the walls; these shelves should hang from the ceiling, by wooden arms, attached firmly before plastering, thus you make all safe from rats.

To those who are so fortunate as to own the houses in which they live, we recommend the month of June, but to renters, the great moving month of May, in New York at least, as the most appropriate time for the following recommendations.

Let the walls and floors be swept thoroughly, on four or five different days, and let a coat of good whitewashing be laid on.

These things should be done once a year, and one day in the week at least, except in mid winter, every opening in the cellar, for several hours, about noon, should be thrown wide; so as to allow as complete a ventilation as possible. Scientific men have forced on the common mind, by slow degrees, the importance of a daily ventilation of our sleeping apartments, so that now, none but the careless or most obtuse neglect it, but few think of ventilating their cellars, although it is apparent that the noisome dampness is constantly rising upwards and pervading the whole dwelling.

Emanations from cellars do not kill in a night, if they did, universal attention would be forced to their proper management, but it is certain, from the very nature of things, that unclean, damp, and mouldy cellars, with their sepulchral fumes do undermine the health of multitudes of families, and send many of their members to an untimely grave; especially must it be so in New York, where the houses are generally constructed in such a manner, that the ordinary access to the cellar, for coal, wood, vegetables, etc. is within the building, and every time the cellar door is opened, the draught from the grating in the street, drives the accumulation of the preceding hours directly upwards into the halls and rooms of the dwelling, there to be breathed over and over again, by every member of the household, thus poisoning the very springs of life, and polluting the whole blood.

With these views we earnestly advise our city readers, as a life-saving thought, in the selection of a dwelling for the ensuing year, to give ten per cent. more for a home which has a model cellar; you will more than save it in doctor's bills, in all probability, to say nothing of taking pills, and drops, and bitters, and gin, from one month's end to another. Let a good cellar determine your choice, rather than the more coveted 'Brown Stone Front," or the locality of Fourteenth Street, Union Square, or Fifth Avenue.

---

BED CURTAINS are unhealthy, because they confine the air around us while we sleep; a canary bird will die in a night, suspended in that situation.

